# Diagnostic Performances of 99mTc-Methoxy Isobutyl Isonitrile Scan in Predicting the Malignancy of Lung Lesions: A Meta-Analysis

**DOI:** 10.1097/MD.0000000000003571

**Published:** 2016-05-06

**Authors:** Shuxin Zhang, Yang Liu

**Affiliations:** From the Department of Thoracic Surgery, Chinese PLA General Hospital (SZ, YL); and Department of Thoracic Surgery, Chinese PLA 309th Hospital (SZ), Beijing, China.

## Abstract

We performed a meta-analysis to evaluate the value of technetium-99m methoxy isobutyl isonitrile (^99m^Tc-MIBI) single photon emission computed tomography (SPECT) in differentiating malignant from benign lung lesions.

The PubMed and Embase databases were comprehensively searched for relevant articles that evaluated lung lesions suspicious for malignancy. Two reviewers independently extracted the data on study characteristics and examination results, and assessed the quality of each selected study. The data extracted from the eligible studies were assessed by heterogeneity and threshold effect tests. Pooled sensitivity, specificity, diagnostic odds ratio (DOR), and areas under the summary receiver-operating characteristic curves (SROC) were also calculated.

Fourteen studies were included in this meta-analysis. The pooled sensitivity, specificity, positive and negative likelihood ratio, and DOR of ^99m^Tc-MIBI scan in detecting malignant lung lesions were 0.84 (95% confidence interval [CI]: 0.81, 0.87), 0.83 (95% CI: 0.77, 0.88), 4.22 (95% CI: 2.53, 7.04), 0.20 (95% CI: 0.12, 0.31), and 25.71 (95% CI: 10.67, 61.96), respectively. The area under the SROC was 0.9062. Meta-regression analysis showed that the accuracy estimates were significantly influenced by ethnic groups (*P* < 0.01), but not by image analysis methods, mean lesion size, or year of publication. Deek funnel plot asymmetry test for the overall analysis did not raise suspicion of publication bias (*P* = 0.50).

Our results indicated that ^99m^Tc-MIBI scan is a promising diagnostic modality in predicting the malignancy of lung lesions.

## INTRODUCTION

Lung cancer is one of the most prevalent and aggressive tumors in the world. The best treatment procedure for patients with a lung lesion depends principally on the probability of cancer for that lesion. Therefore, the accurate diagnosis of lung lesions is crucial not only for early detection of malignancies, but also to avoid unnecessary surgery for benign lesions.^1^

Various noninvasive approaches have been tried to differentiate benign from malignant lung lesions. Conventional imaging modalities, such as chest computed tomography and magnetic resonance have a limited diagnostic value since their interpretation was based on lesion size.^2^ Functional nuclear imaging methods, that demonstrate the metabolic properties of a lesion, have been applied for the identification of lung cancer. Several studies have shown that ^18^F-fluorodeoxyglucose positron emission tomography(FDG-PET) has been widely accepted in clinical practice due to its good test performance in identifying lung cancer.^3,4^ FDG-PET scanner is helpful but the high cost and limited availability have restricted its clinical use.^5^ Additionally, other studies have questioned the role of FDG-PET in detecting lung cancer. Several inflammatory diseases (tuberculosis, inflammatory pseudo tumor, pneumonia, and abscess) have been associated with the high uptake of glucose.^6^ In addition, some well-differentiated malignancies such as bronchioalveolar carcinoma and carcinoids may not be visualized by FDG-PET.^7,8^

Consequently, single photon emission computed tomography (SPECT) has been proposed as a feasible alternative method in lung cancer imaging. Commonly used tracers in lung cancer imaging by SPECT include ^99m^Tc-depreotide, talium-201 chloride (^201^Tl), and technetium-99m methoxy isobutyl isonitrile (^99m^Tc-MIBI).^9–11^ Encouraging results in lung cancer detection have been obtained with ^99m^Tc-MIBI, a lipophilic cation widely used as a tracer for myocardial perfusion imaging.^12,13^^99m^Tc-MIBI has been emphasized in clinical use due to its shorter half-life, improved spatial resolution, lower cost, and easy availability.^14,15^ The bio-distribution of ^99m^Tc-MIBI is characterized by rapid blood clearance and consequently by early uptake by target organs.^16^ Early imaging of malignancies, at 10 minutes after intravenous injection of ^99m^Tc-MIBI, is satisfactory.^17,18^ However, published articles about test performance of ^99m^Tc-MIBI scan in distinguishing malignancy from benign lung lesions are discordant and a meta-analysis on this topic was lacking. The purpose of the present study is to systematically investigate the efficacy of ^99m^Tc-MIBI scan in detection of malignant lung lesions.

## MATERIALS AND METHODS

### Search Strategy

This meta-analysis followed the Preferred Reporting Items for Systematic Reviews and Meta-analyses criteria.^19^ A systematic computer literature search of PubMed and Embase databases was conducted to identify relevant articles published until August 20, 2015 concerning the assessment of ^99m^Tc-MIBI SPECT in patients with lung lesions suspicious for malignancy. Our search algorithm was based on a combination of the following terms: (1) “MIBI” or “sestamibi” or “methoxyisobutylisonitrile”; and (2) “lung” in this search. All searches were limited to human studies without language restriction. Additionally, to enlarge our search, references of the relevant studies and review articles were also manually checked. We directly contacted the corresponding author for more detailed information if the articles provided insufficient information.

### Study Selection

The inclusion criteria for this meta-analysis were as follows: (1) ^99m^Tc-MIBI SPECT was performed to identify and characterize the suspected lung cancer; (2) studies in which 2 × 2 tables could be extracted and the reported data were sufficient to calculate true positive (TP), false positive (FP), false negative (FN), true negative (TN), sensitivity (SEN), specificity (SPE), positive likelihood ratio (LR+), negative likelihood ratio (LR−) values and diagnostic odds ratio (DOR); (3) the study enrolled at least 10 participants with benign or malignant lesions; (4) pathology and/or close clinical and/or radiological follow-up were used as the reference standard; and (5) no data overlap (if studies had the overlapping data, only the study with the most complete article was included in the final analysis). Studies were excluded based on the following criteria: (1) studies in which 2 × 2 tables could not be extracted, (2) previous therapy before ^99m^Tc-MIBI scan, and (3) animal studies, case reports, abstracts, review articles, letters, editorials, comments, and conference proceedings.

Two investigators independently reviewed the titles, abstracts, and full text (if available) of potentially relevant articles, applying the above-mentioned inclusion and exclusion criteria. Any differences were resolved by consensus.

### Data Extraction

We constructed the 2 × 2 contingency tables according to the reference standard. Information extracted included baseline characteristics (authors, year of publication, ethnic group), study design (prospective or retrospective), patients’ characteristics (sample size, mean size of lung lesion, mean age, gender), as well as technical characteristics of ^99m^Tc-MIBI SPECT (image analysis method, reference standard).

### Quality Assessment

The same 2 independent reviewers assessed the methodological quality of the selected studies using a revised version of Quality Assessment Tool for Diagnostic Accuracy Studies version 2 (QUADAS-2).^20^ This modified tool is composed of 4 domains related to “Risk of bias” judgment. For 3 of the 4 domains related to concerns of applicability. The disagreements between 2 investigators were resolved by means of consensus.

### Statistical Analysis

The recommended standard methods for diagnostic performance of meta-analysis were used.^21,22^ Heterogeneity in studies caused by a threshold effect was tested using the Spearman rank test. A notable correlation indicated a threshold effect, with *P* < 0.05. In addition, the presence of heterogeneity among different studies was tested using chi-squared tests and the inconsistency index (*I*^2^). Notable heterogeneity was defined as *P* < 0.1 or *I*^2^ > 50%. If these studies showed sufficient clinical homogeneity, the statistical pooling of the data was performed using a fixed-effects model; otherwise, a random-effects model was used.^23,24^ The pooled SEN, SPE, LR+, LR−, and DOR were presented with 95% confidence intervals (95% CI). The observed sensitivity and specificity for ^99m^Tc-MIBI test performance are displayed using forest plots.

A summary receiver-operating characteristic curve (SROC) was obtained for selected studies and area under the curve (AUC) was calculated to assess the overall accuracy of ^99m^Tc-MIBI imaging. AUC values <50% would indicate that the diagnostic test has no test performance. AUC values ranging from 51% to 70%, from 71% to 90%, and >90% indicated low, moderate, and high diagnostic performance, respectively.

Subsequently, subgroup analyses were also performed using a more homogeneous group of studies.^25^ Subgroups including more than 3 studies would be constructed. Differences between subgroups were assessed through test of interaction.^26^ In this study, we used meta-regression analysis to assess potential heterogeneity source. Anticipated sources of heterogeneity (mean size of lung lesion, year of publication, ethnic group, and image analysis method) were included in the meta-regression.

The above mentioned statistical analyses were performed using Meta-Disc (version 1.4) software package.^22^ Publication bias analysis was performed using Stata 12.0 (Stata Corp, College Station, TX).

## RESULTS

### Study Characteristics and Quality Assessment

**FIGURE 1:**
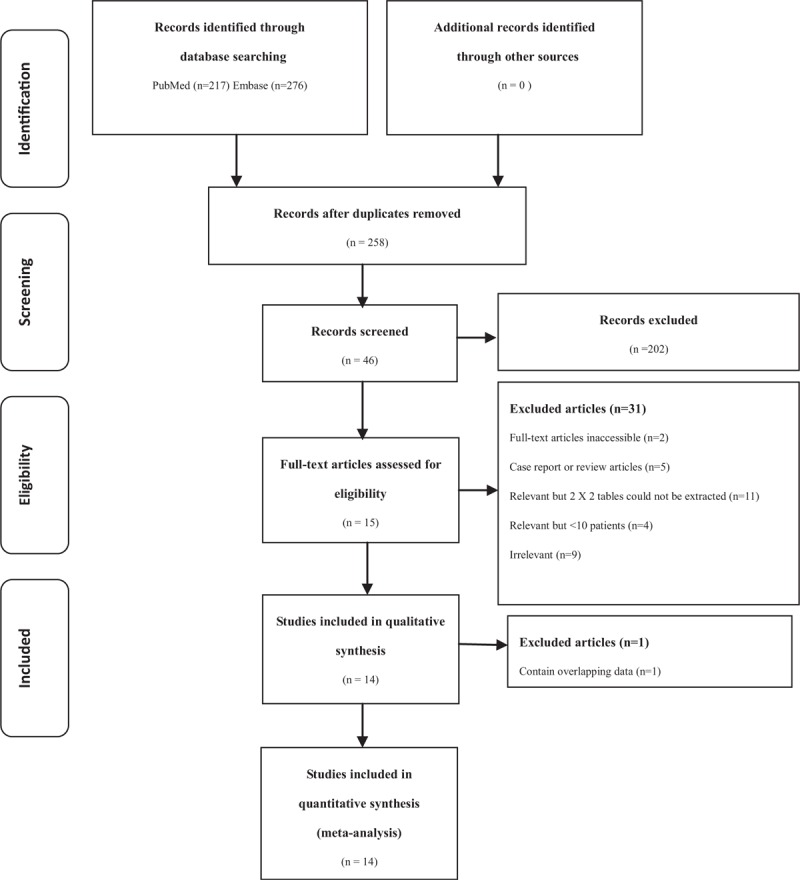
Flow diagram of the study selection process.

The literature search process is demonstrated as a flowchart in Figure 1. Of the 15 articles deemed eligible for inclusion,^10,12,27–30,32–39^ one pair of article by Nikoletic et al^31,32^ had overlapping data; thus, only the latest article^32^ was included. Consequently, the final review comprised 14 studies. The characteristics of these eligible studies are outlined in Table 1. The sample size of the 14 studies ranged from 23 to 116; a total of 688 eligible patients were included in this meta-analysis.

**TABLE 1:**
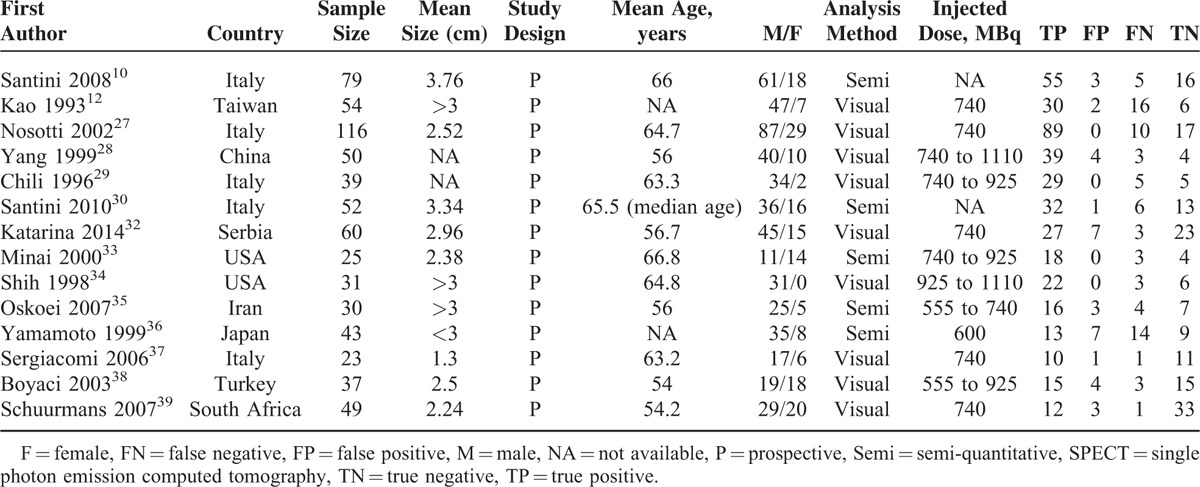
Characteristics of Studies Included in the Meta-analysis of ^99m^Tc-MIBI Scan in Detection of Malignant Lung Lesions

All the 14 studies were prospective cohort studies. Image analysis was visual in 9 studies and semi-quantitative in 5. All 14 included studies had pathology and/or clinical and/or radiological follow-up as the reference standard. The injected dose ^99m^Tc-MIBI ranged from 555 to 1110 MBq. None of these studies reported any adverse events.

We used the “QUADAS-2” quality assessment tool to evaluate the individual study. Table 2 shows the risk of bias and concerns about the applicability of the 14 selected articles in this study. Overall, the studies included in this meta-analysis have shown satisfactory methodological quality according to QUADAS-2.

**TABLE 2:**
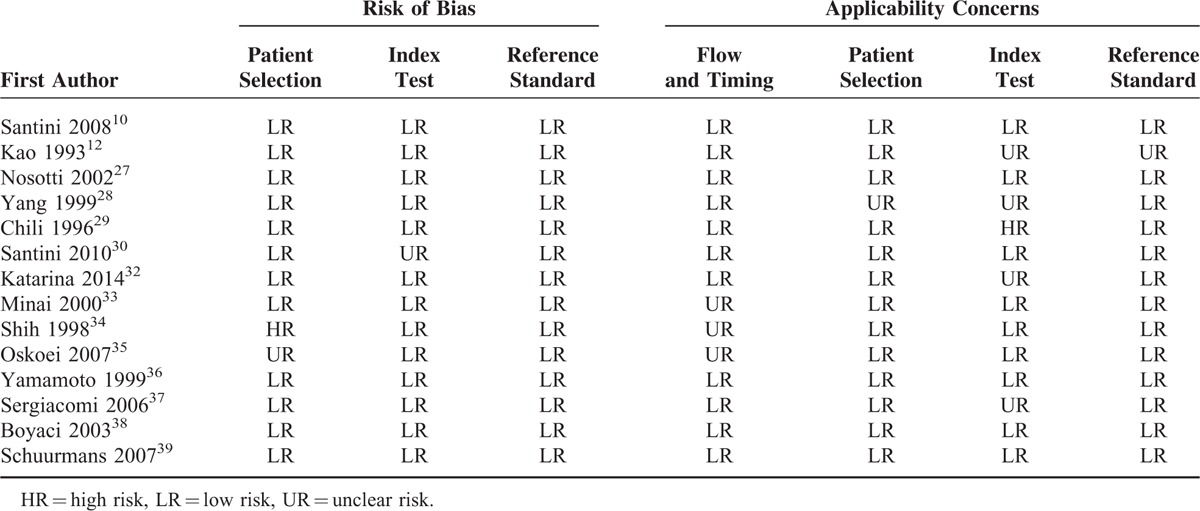
Summary of the Quality Assessment Tool for Diagnostic Accuracy Studies Version 2 (QUADAS-2) Assessment

### Quantitative Analysis

**FIGURE 2:**
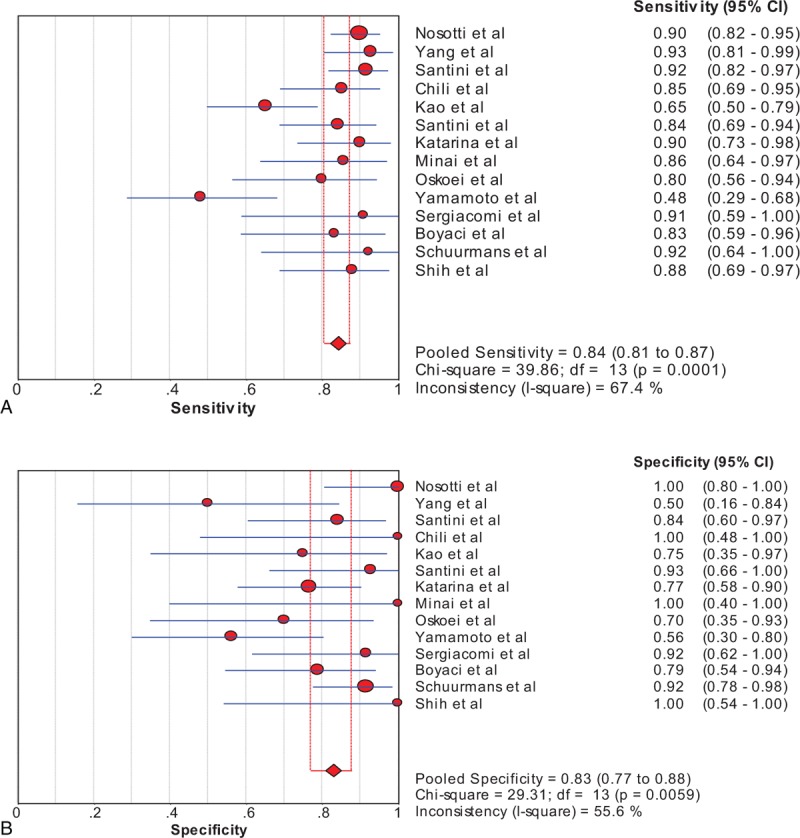
Forest plot of the sensitivity (a) and specificity (b) of the meta-analysis. The sensitivity and specificity of each study are represented by circles, with the 95% CI of each represented by the horizontal line through each circle. The pooled sensitivity and specificity for this meta-analysis are represented by diamonds. CI = confidence interval, df = degrees of freedom.

**FIGURE 3:**
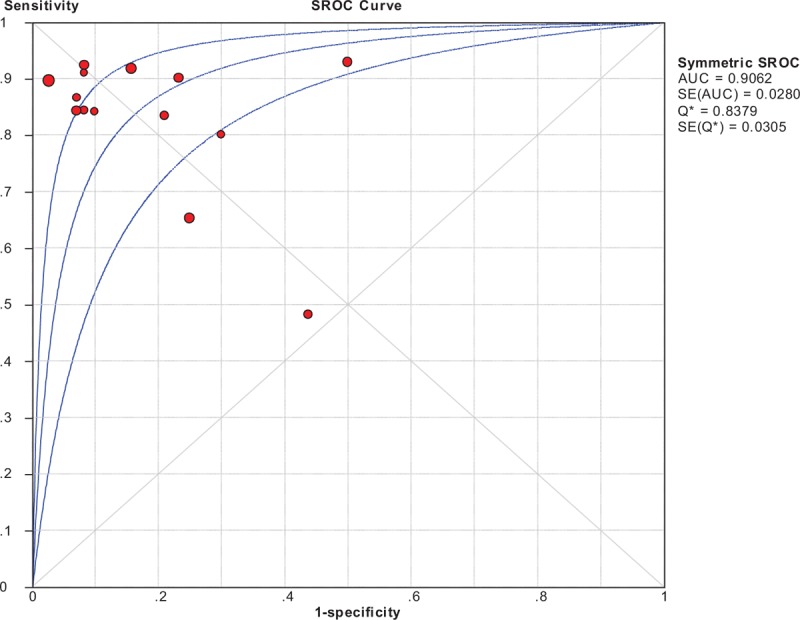
Summary receiver-operating characteristic curve of ^99m^Tc-MIBI scan in detection of malignant lung lesions. AUC = area under the curve, SE = standard error, SPECT = single photon emission computed tomography, SROC = summary receiver-operating characteristic. The middle blue line represents the SROC curve and the other 2 represent confidence intervals. Each red dot in the SROC plot represents a separate study in this meta-analysis. Q^∗^ index represents the point on the SROC at which SEN and SPE are equal.

A total sample size of 688 patients with suspected lung cancer was included in our meta-analysis. In the overall studies, SEN had a wide distribution, ranging from 0.48 to 0.93, with all but one^36^ >0.6. SPE ranged between 0.50 and 1.0, with all but 2^28,36^ >0.7. In this meta-analysis the Spearman correlation coefficient was −0.24 (*P* = 0.41), suggesting that a significant threshold effect does not exist in accuracy evaluations in the included studies. The test performance was summarized using a random-effects coefficient binary regression model for the significant heterogeneity observed in our studies. The diagnostic performance values of ^99m^Tc-MIBI SPECT in the 14 studies included in the review are presented in Figure 2. The pooled SEN, SPE, LR+, LR−, and DOR were 0.84 (95% confidence interval [CI]: 0.81, 0.87), 0.83 (95% CI: 0.77, 0.88), 4.22 (95% CI: 2.53, 7.04), 0.20 (95% CI: 0.12, 0.31), and 25.71 (95% CI: 10.67, 61.96), respectively. The area under the SROC was 0.91 and the SROC curves are shown in Figure 3.

Among the 5 studies reporting average lesion diameter of greater than 3 cm, the pooled SEN, SPE, LR+, and LR− were 0.82(0.76, 0.87), 0.841(0.72, 0.93), 4.13(2.31, 7.38), and 0.21(0.11, 0.39), respectively, with an AUC under the SROC of 0.90. Seven studies with an average lesion diameter of less than or equal to 3 cm had the similar diagnostic performance. The AUC showed no difference between the 2 subgroups (mean size of lesion ≤ or >3 cm in diameter) (*P* = 0.91).

Ethnic group was significantly associated with diagnostic efficacy. Three studies were performed in Asian group. The pooled SEN, SPE, LR+, and LR− were 0.71(0.62, 0.79), 0.59(0.41, 0.76), 1.54(0.98, 2.43), and 0.47(0.20, 1.07), respectively, and an AUC under the SROC of 0.67. The other 10 studies were performed in Caucasian patients. The AUC differed significantly between Asian group and Caucasian group (*P* < 0.01).

Subgroup analyses by year of publication (1993–1999 versus 2000–2006 versus 2007–2014) and image analysis methods (visual versus semi-quantitative analysis) were also performed. Results of subgroup analyses are demonstrated in Table 3.

**TABLE 3:**
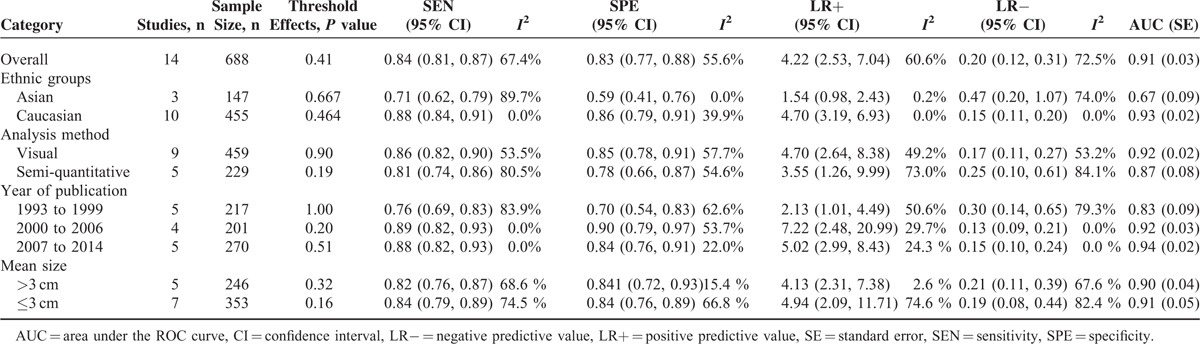
Subgroup Analyses of Diagnostic Accuracy Variables

### Heterogeneity Analysis and Publication Bias

There was notable heterogeneity in the studies included in the summarized analysis. Meta-regression analysis showed that the accuracy estimates were significantly influenced by the ethnic group (Asian group versus Caucasian group, *P* < 0.01), but not by image analysis methods, mean lesion size, or year of publication.

**FIGURE 4:**
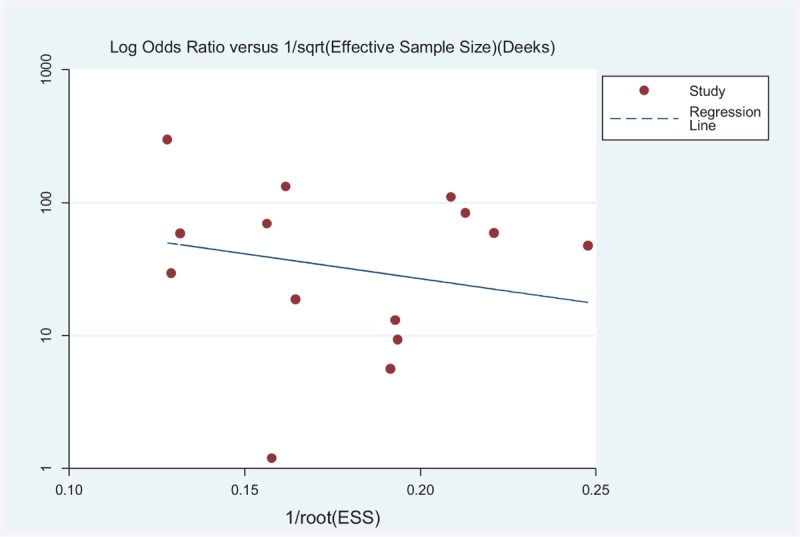
Funnel plot of publication bias of studies included.

Deek funnel plot asymmetry test for the overall analysis did not raise suspicion of publication bias (*P* = 0.50) (Figure 4).

## DISCUSSION

To our knowledge, this is the first study to systematically evaluate the test performance of ^99m^Tc-MIBI SPECT for diagnosis of lung cancer. The pooled sensitivity and specificity were at around 0.8, and the AUC under the SROC for the overall analysis was 0.91, indicating that ^99m^Tc-MIBI SPECT has moderately good overall diagnostic accuracy for identifying lung cancer. Furthermore, the DOR is a single summary measure of the test power, independent of prevalence.^40^ In simple terms, the higher the DOR is, the better test performance the test has.^41^ The overall DOR was 25.71 for this meta-analysis. In addition, a good diagnostic test may have LR+ greater than 10, LR− less than 0.1 to have a greatest test performance.^41^ Nevertheless, this review gave an overall LR+ of 4.22 and LR− of 0.20 indicating that ^99m^Tc-MIBI SPECT can neither confirm nor exclude nodal metastasis in lung cancer. In general, ^99m^Tc-MIBI SPECT is a useful imaging modality in clinical practice.

The test performance did not differ significantly between 2 subgroups according to their mean size of lesion (≤ or >3 cm in diameter), as demonstrated by our sub-analysis. Our result was in concordance with the findings of Santini et al^10^ and Nosotti et al,^27^ who reported that tumor delectability by ^99m^Tc-MIBI SPECT is independent of the lesion size. That is, it does not mean that a larger lesion will be easier to be identified on ^99m^Tc-MIBI SPECT.

Image analysis methods for detecting lung cancer vary considerably in these studies. Visual analysis was performed in 9 of 14 studies. Semi-quantitative analysis was performed in the other 5 studies. However, the subgroup analysis showed no difference in diagnostic performance between the 2 methods. Up to now, there is no standard criterion reported for identifying lung cancer on SPECT. The visual assessment of images is crucially influenced by the experience of the image readers. Semi-quantitative analysis method, such as region of interest analysis or the lesion-to-background ratio, may help standardize the image evaluation.^33,42^ Thus, further studies are needed to put forward the ideal criteria for interpreting SPECT scans.

Although this meta-analysis showed promising results for the diagnostic accuracy of ^99m^Tc-MIBI SPECT in lung cancer detection, the outcomes should be interpreted with caution due to several limitations. Firstly, the studies varied in year of publication, sample size, continuity of patients enrolled, and ethnic groups as well as lesion size. Besides, ^99m^Tc-MIBI SPECT images were performed under variable conditions, including tracer dose, image analysis methods, the interval time between tracer injection and scanning.

Secondly, it is impossible for us to identify all studies of ^99m^Tc-MIBI SPECT for lung cancer diagnosis, especially unpublished studies. Since articles reporting significant results are more likely to be published than those reporting non-significant results, publication bias is a major concern in meta-analysis. However, the Deek funnel plot asymmetry test for the overall analysis did not raise suspicion of publication bias. In addition, we adopted rigid inclusion criteria and we selected only articles that included at least 10 patients who performed MIBI imaging for lung lesions, which may bring about selection bias.

Thirdly, it was not clear whether SPECT or PET is superior in differentiating malignant from benign lesions. Two recent published meta-analyses^7,43^ were performed to evaluate the diagnostic accuracy of FDG-PET for detecting lung cancer with a sensitivity of 94% to 96% and specificity of 78% to 86%. However, a direct comparison between PET and SPECT is in the absence. Only 2 of the studies^30,39^ compared SPECT with PET included in our meta-analysis, but the results were generally inconclusive. According to Santini et al,^30^^99m^Tc-MIBI SPECT was similar to FDG-PET in the detection of lung malignancies and represents an alternative if PET was not available. Finally, a further interesting point of discussion is a comparison of PET and ^99m^Tc-MIBI SPECT to estimate the value of these modalities in differentiating malignant and benign lung lesion.

## CONCLUSIONS

In conclusion, this meta-analysis showed that ^99m^Tc-MIBI SPECT scan had moderately good diagnostic performance in predicting the malignancy of lung lesions. Despite of the limitations described above, the non-invasiveness, low cost, and the easy availability of ^99m^Tc-MIBI SPECT make it a reliable diagnostic tool in the evaluation of lung lesions.
